# Transapical combined transcatheter aortic valve-in-valve implant and ascending aorta endovascular repair

**DOI:** 10.1093/icvts/ivac002

**Published:** 2022-01-24

**Authors:** Andrea Agostinelli, Alan Gallingani, Bruno Borrello, Francesco Nicolini

**Affiliations:** Cardiac Surgery Unit, Parma University Hospital, Parma, Italy

**Keywords:** Transcatheter aortic valve implant valve-in-valve, Ascending aorta thoracic endovascular aortic repair, Transapical, Transcatheter aortic valve implantation

## Abstract

We report the case of a 64-year-old patient who previously had an aortic valve replacement with a stentless aortic valve and an ascending aorta replacement for a DeBakey type II aortic dissection. The patient was referred to us for symptomatic aortic regurgitation related to bioprosthesis degeneration and a pseudoaneurysm at the distal anastomotic site of the vascular graft. Due to the presence of several comorbidities, the patient had a combined transapical transcatheter aortic valve-in-valve implant and an ascending aorta endovascular repair.

## INTRODUCTION

A transapical transcatheter aortic valve implant (TAVI) is a well-established alternative approach to the treatment of an aortic valve in patients with unsuitable peripheral access [1]. On the basis of their experiences with TAVI, many authors have also reported the safety and effectiveness of the transapical approach in thoracic endovascular aortic repair (TEVAR) procedures in all cases with hostile iliac-femoral axes [2]. Thoracic endovascular repair of the ascending aorta proved a safe and effective alternative procedure for patients deemed unsuitable for open surgery.

We report the successful treatment of a patient using a combined transapical transcatheter aortic valve-in-valve implant and an ascending aorta endovascular repair.

## CASE REPORT

**Figure 1:**
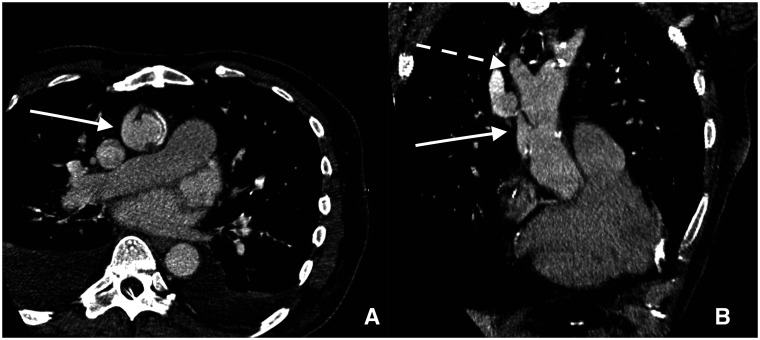
Preoperative imaging. (**A, B**) Multiplanar computed tomography angiography reconstruction in which the pseudoaneurysm at the distal anastomotic site (white arrow) and the brachiocephalic trunk origin (white dashed arrow) are clearly visible.

A 64-year-old man previously had an aortic valve replacement with a stentless 25-mm Freestyle aortic root prosthesis (Medtronic, Inc., Minneapolis, MN, USA) and an ascending aorta replacement for a DeBakey type II aortic dissection with a 28-mm Hemashield vascular graft (Getinge, Gothenburg, Sweden) was admitted to a spoke hospital with severe dyspnoea. Transthoracic echocardiography showed severe regurgitation due to degeneration of the bioprosthesis. A computed tomography (CT) scan revealed a 15- × 32-mm pseudoaneurysm at the level of the distal anastomosis of the vascular graft. The neck of the pseudoaneurysm was located 40 mm from the origin of the brachiocephalic trunk and 45 mm from the origin of the left main coronary artery (Fig. 1A and B). The diameter of the mean ascending aorta distal to the vascular graft was 29.8 mm.

The patient had several comorbidities: a history of myasthenia gravis treated with pyridostigmine, methotrexate and oxygen therapy at home; a previous myocardial infarction; severe peripheral artery occlusive disease involving the aorto-iliac-femoral axes and the carotid arteries and multiple vertebral collapses. A previous major cerebral accident left him with left-sided residual hyposthenia.

The case was evaluated by a multidisciplinary heart team that included a cardiac surgeon, a cardiologist, a radiologist and an anaesthesiologist. An open surgical operation was ruled out, and the patient was scheduled for a combined transapical TEVAR and TAVI.

**Figure 2:**
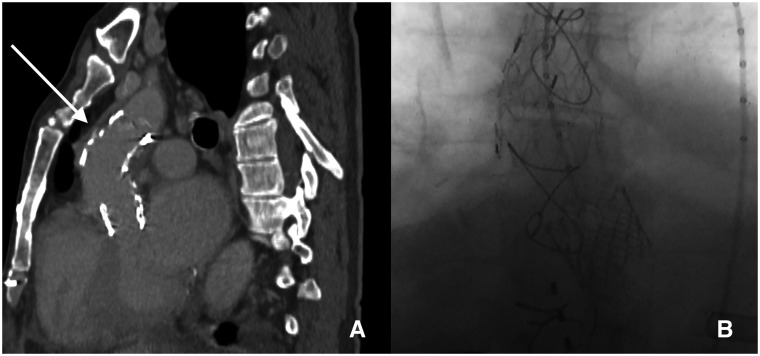
Postoperative imaging. (**A**) Computed tomography angiography scan revealing correct stent graft position and pseudoaneurysm exclusion (white arrow). (**B**) Final result: X-ray view.

The procedure was carried out in a cardiac surgery operating theatre through a left anterior minithoracotomy. Under ventricular pacing, we implanted a Gore Excluder 32- × 45-mm aortic cuff (W. L. Gore & Associates, Flagstaff, AZ, USA) and subsequently, an Edwards Sapien Ultra 26-mm aortic bioprosthesis (Edwards Lifescience Corp., Irvine, CA, USA); for both TEVAR and TAVI, the 21 Fr Edwards Certitude sheath was inserted into the surgically isolated left ventricular apex and positioned below the bioprosthetic aortic valve. The Gore Excluder cuff was released into the ascending aorta with a distal landing zone 2 cm from the origin of the brachiocephalic trunk and proximal landing zones 3 and 3.7 cm from the origin of the left main coronary artery and the right coronary artery, respectively. After haemodynamic stability was restored, the Sapien 3 Ultra valve was released (Fig. 2B and see Video 1).

The postoperative intensive care unit stay was 24 h; the patient was discharged uneventfully on postoperative Day 6.

A predischarge echocardiogram showed the correct valve position and function and the absence of paravalvular leaks. A postoperative CT scan demonstrated complete exclusion of the pseudoaneurysm and no endoleaks (Fig. 2A). At the 6-month follow-up visit, the patient was healthy. A CT scan confirmed complete exclusion of the pseudoaneurysm and the absence of endoleaks.

## DISCUSSION

The transapical approach for TAVI in patients with a hostile peripheral access is a well-established procedure [1]. On the basis of this experience, we began to apply the same approach for emergency/urgent TEVAR [2]. TEVAR has also proved to be a feasible option for treatment of the ascending aorta, especially in high-risk patients [3].

Valve-in-valve TAVI has been described in the literature as a safe, feasible procedure for treating a degenerated stentless bioprosthesis with pure regurgitation [4]. On the CT scan reconstruction, the aortic valve area was 398 mm^2^; considering the absence of detectable calcification, we chose an oversized 26-mm balloon-expandable valve to obtain a perfect seal.

We rejected the transfemoral access because of severe peripheral artery occlusive disease. Due to the short distance and the better delivery angle, we also believed that the transapical procedure would be less technically demanding and could offer more stability during valvular and vascular aortic graft deployment.

To our knowledge, there is only 1 reported case (Allen *et al.* [5]) of using the same smart pathway, but that was in a native aortic valve and ascending aorta.

Although the present report presents a single case, we wish to highlight the importance for the future of personalized medicine of a multidisciplinary team approach and the use of a wide range of surgical techniques that allow the successful application of true patient-oriented approaches.
